# “Slow” skeletal muscles across vertebrate species

**DOI:** 10.1186/s13578-015-0054-6

**Published:** 2015-11-14

**Authors:** Victor M. Luna, Eriko Daikoku, Fumihito Ono

**Affiliations:** Division of Integrative Neuroscience, Department of Psychiatry, New York State Psychiatric Institute, Columbia University, New York, NY 10032 USA; Department of Molecular Physiology, Osaka Medical College, 2-7 Daigaku-machi, Takatsuki, Osaka 569-8686 Japan; Laboratory of Molecular Physiology, National Institute on Alcohol Abuse and Alcoholism, National Institutes of Health, Bethesda, MD 20892 USA

**Keywords:** Zebrafish, Muscle, Slow fiber, Fast fiber, Acetylcholine receptor, Synapse

## Abstract

Skeletal muscle fibers are generally classified into two groups: slow (type I) and fast (type II). Fibers in each group are uniquely designed for specific locomotory needs based on their intrinsic cellular properties and the types of motor neurons that innervate them. In this review, we will focus on the current concept of slow muscle fibers which, unlike the originally proposed version based purely on amphibian muscles, varies widely depending on the animal model system studied. We will discuss recent findings from zebrafish neuromuscular junction synapses that may provide the framework for establishing a more unified view of slow muscles across mammalian and non-mammalian species.

## Introduction: overview of skeletal muscle classification systems

It is widely accepted that the skeletal muscle system in vertebrates consists mainly of two types of muscle fibers: slow and fast, with fast fibers being further classified into two or three subtypes. The concept of muscles being slow and fast originally arose from physiological studies of frog muscles in the 19th century. Initially, a single muscle fiber was assumed to exhibit one of two contraction properties depending on the experimental conditions, namely “tonic” or “phasic” [2]. In “tonic” states, fibers displayed prolonged contractions in response to a nerve stimulus [2]. In contrast, in “phasic” states, fibers displayed rapid twitches. It was not until the 1950s that Stephen Kuffler’s lab showed that these two states actually arose from two distinct populations of fibers and that individual muscles had different characteristics based on the variable proportions of each fiber type [3]. Tonic fibers have since been called slow fibers, a term also used to refer to mammalian fibers with several similar cellular properties.

However, slow fibers in frog muscles are quite distinct from mammalian slow fibers. One important distinction between tonic and phasic fibers in frogs was their ability to fire action potentials: tonic fibers did not generate spikes while phasic fibers propagated them. In contrast, mammalian slow fibers do fire action potentials. Furthermore, frog tonic muscles are distinguished from phasic muscles by their lack of “twitches”. Mammalian slow muscles are capable of twitching and are in fact called “slow-twitch” muscles (as opposed to “fast-twitch” muscles). Thus the common use of the term “slow muscle” to refer to both non-mammalian and mammalian is a source of much confusion in previous and current studies of skeletal muscles.

In parallel with classical physiological methods, chemical techniques were also employed to characterize muscle fibers based on biochemical properties related to energy metabolism. Fibers with the alkali-labile ATPase were labeled “type I”, while those with the acid-labile ATPase system were termed “type II” [4]. Several proteins were subsequently revealed to be specific for type I or type II muscle fibers. For instance, the myosin heavy chain gene has some isoforms which are preferentially found in either type I or type II fibers [5]. These isoforms, rather than the originally proposed ATPase types, provide more perspicuous bases for defining type I and type II fibers. Slow and fast fibers in chick, for example, do not exhibit differences in ATPases [6]. Additionally, proteins related to the Ca^2+^ cycle, such as parvalbumin and SERCA, also show fiber specific distribution [7]. Over time, studies have shown that slow fibers and type I fibers shared many identical characteristics. These findings have since led to the interchangeable use of each term.

Overall, slow fibers are associated with the following properties: slow contraction, oxidative metabolism with low ATPase activity, and resistance to fatigue [7]. Due to the larger amount of myoglobin contained in them, type I fibers are sometimes called red fibers while type II fibers are called white fibers. However, the contraction properties and oxidative metabolic capabilities of slow muscles are actually highly variable across species [2, 8–14].

Focusing on the molecular mechanism of fiber differentiation may provide a clearer view of fiber type classification. To date, studies utilizing molecular biology techniques have determined the genetic mechanisms controlling the identity of each muscle fiber type. For instance in fish, *Prdm1* is a transcription factor that activates slow muscle specific genes while suppressing fast muscle genes. *Prdm1* acts by suppressing *sox6*, a repressor of slow muscle specific genes [15]. Remarkably, when one such control gene—transcriptional co-activator *PGC*-*1*—was ectopically expressed, type II fibers were converted to type I fibers in mice [16]. However, the mechanism and molecular players are again quite variable across species. *Prdm1* in mouse, for example, seems to work in a different manner and its role in myogenesis remains unclear [15].

Type I and type II muscle fibers are now extensively studied in human physiology, particularly in the context of sports science. For instance, endurance training increases the proportion of type I fibers relative to type II fibers [17], while sprint training increases the cross-sectional area of type II fibers [18]. In addition, genetic factors influencing the divergent properties of the two fiber types have received considerable attention in both academia and industry [19, 20]. It is therefore perplexing that the fundamental concept of what a “slow” fiber is remains controversial. In the following sections, we will attempt to address this issue by discussing how findings in zebrafish could provide a more unified definition of “slow” muscle.

## Motor neuron innervation of fast versus slow muscle

**Fig. 1:**
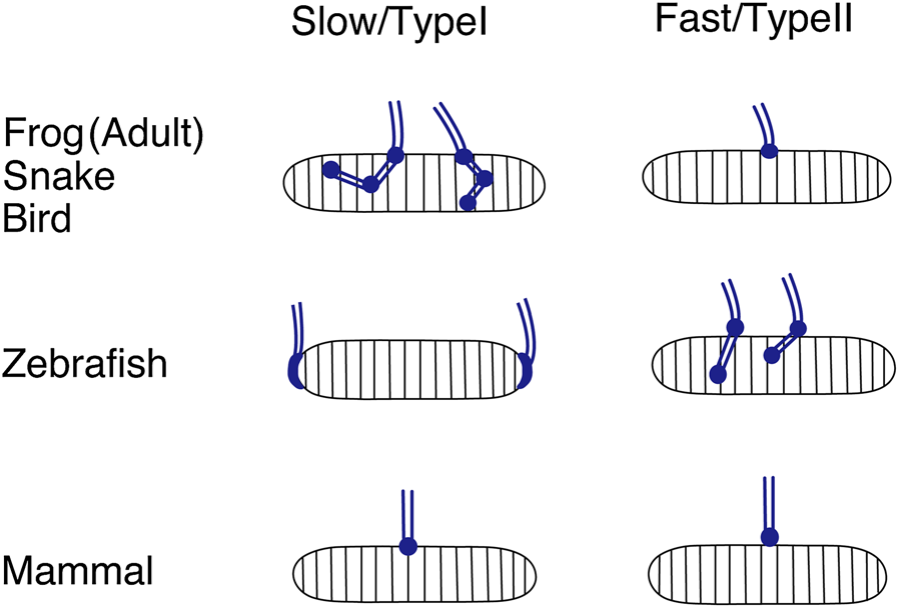
Schemas depicting motor neuron axons (*blue*) innervating individual muscle fibers. Note the differences in synaptic distribution between fiber types and animals

Based on early studies of frogs, snakes, and birds, the anatomical pattern of motor neuron innervation was once considered a viable criterion for distinguishing between slow and fast muscles [21]. The innervation of fast muscles was said to be “en plaque”: the classic endplate morphology, with discrete and focal nerve endings [10] (Fig. 1). In contrast, the innervation of slow muscles was said to be “en grappe”: diffuse, multi-terminal, and distributed (Fig. 1).

However, studies examining a wider variety of species have shown that fast and slow fibers exhibit highly variable patterns of innervation, making them much more difficult to generalize than initially expected. For example, both fast and slow muscles have multiple nerve endings in fish and therefore neither corresponds to the classic definition of “en plaque” [22]. Additionally, amphibian tadpoles (before metamorphosis) and fish muscles are innervated at their myoseptal regions [23]. These synapses are located at the boundaries between body segments at the distal edges of individual muscle cells (Fig. 1). Interestingly, it was recently shown that in zebrafish myospetal innervation is actually restricted to slow muscle fibers [24].

In mammalian type I muscles, synapses are formed “en plaque” and not “en grappe”, again deviating from the classical observations in frog muscles [25]. A notable exception are the extraocular muscle fibers which appear to have en grappe-like synapses [26, 27]. Thus, because of its high variability and species-specific nature, it became clear that motor neuron innervation patterns could not be used as a reliable criterion for categorizing muscle fiber types.

However, in the 1950s, several studies suggested that distinct sets of motor neurons innervated slow and fast muscles. These experiments were performed in frogs, where it was found that small nerves innervated slow fibers and large nerves innervated fast fibers [3]. Similarly in fish, slow muscle fibers are innervated by small secondary motor neurons, while fast muscle fibers are innervated by both these neurons and the much larger primary motor neurons [28]. Each motor neuron type is involved in different kinds of swimming behavior, with secondary motor neurons being active at the lowest swimming frequencies, while primary motor neurons are recruited at increasing locomotory speeds [29]. To this end, secondary motor neurons fire action potentials intermittently (chattering) or in bursts, while primary motor neurons spike tonically [30].

Furthermore, studies that have investigated the effect of motor neuron firing on mammalian muscle fiber identity have shown that infrequent (10 Hz frequency) electric stimulation of fast muscle fibers significantly prolonged their contraction and relaxation times [12]. Moreover, switching motor neuron innervation between fiber types results in a similar conversion of contraction and relaxation speeds such that slow fibers behaved liked fast fibers and vice versa [9].

## Zebrafish skeletal muscle fibers

**Fig. 2:**
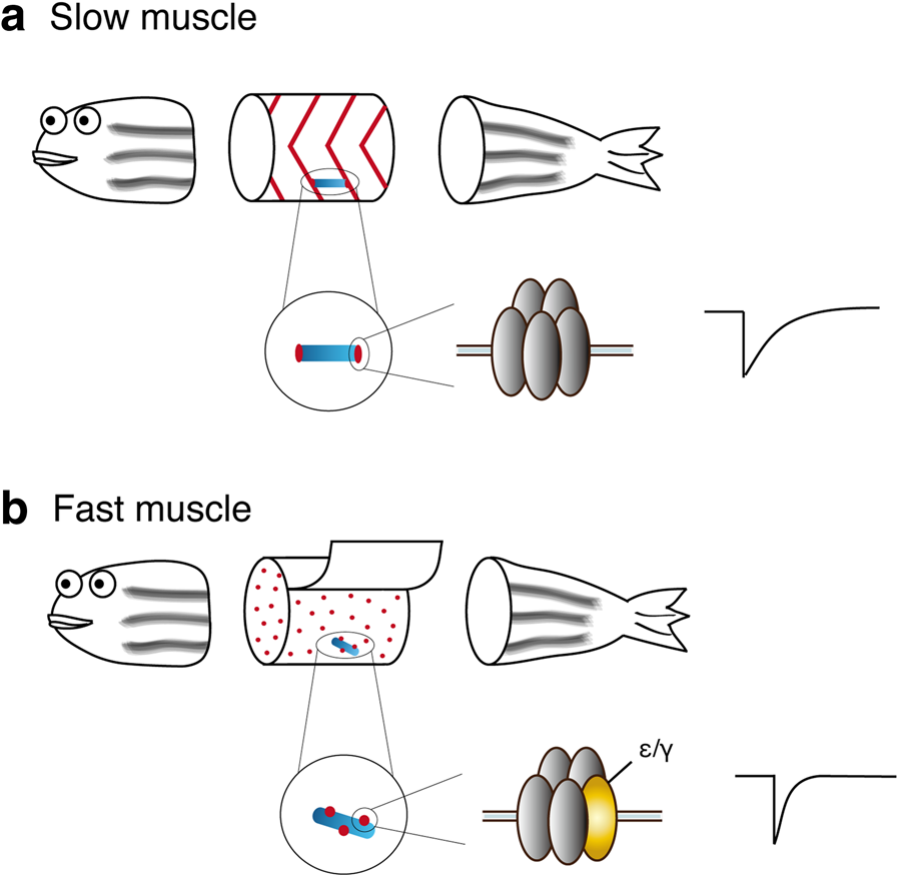
**a** Slow fibers (a single fiber shown in *blue*) are found directly underneath the skin. Synapses (shown in *red*) are found at the chevron-shaped body segment boundaries. At the individual fiber level, these synapses are at the distal edges. AChR pentamers found in these synapses do not include the λ or ε subunit, and generate currents with slow decay kinetics. **b** Fast fibers (a single fiber shown in *blue*) are located in layers deeper than slow fibers. Synapses (*red*) can be observed as round spots in each fiber. AChRs contain λ or ε subunit (*yellow*) and generate fast synaptic current kinetics

The zebrafish is an animal model system widely used for biomedical research. Anatomical, developmental, physiological, and molecular information about this system makes it ideal for studying muscle fiber types. In developing zebrafish embryos, slow fibers initially differentiate as adaxial muscles in the innermost layer of the skeletal muscle near the notochord around 13 h postfertilization (hpf), then migrate outward as the animal develops [31]. They then settle and form a single superficial layer of muscle fibers directly underneath the skin (Fig. 2). In contrast, fast fibers form multiple deeper layers, thereby constituting the bulk of the trunk.

Early in development, motor neuron axons exit the spinal cord near the center of the body segment and form “en passant” synapses with muscle cells along their way [32]. Motor neuron projections branching from the main trunk form synapses on fast muscle fibers [28]. Once near the body surface, axons run along the boundaries of the body segments forming myoseptal synapses in slow muscles [33].

Synapses formed on slow and fast muscle have strikingly different electrophysiological properties. In both intracellular and extracellular recordings, the decay of miniature endplate currents (mEPCs) in slow fibers has a longer time constant than in fast fibers. This allows mEPCs to readily traverse the highly coupled network (via gap junctions) of slow muscle fibers [34]. It needs to be noted, however, that this electrical network may be specific to the larval stage and may not apply to adult slow muscles [35].

Recent studies have shown that the differences in mEPC kinetics between slow and fast muscle cells are a result of the distinct molecular compositions of acetylcholine receptors (AChRs) in each fiber type [36] (Fig. 2). AChRs in fast fibers are composed of α, β, δ subunits and an additional ε or γ subunit. Those in slow fibers lack the ε or γ subunit and are comprised of only α, β and δ subunits [37]. This difference in subunit composition was highlighted in a recent study of *love sofa*, a zebrafish mutant in which only slow fibers had functional AChRs at NMJs [24].

## Why have special synapses in zebrafish slow muscle?

Zebrafish slow and fast muscle are involved in different types of swimming behavior [22, 38–40]. Slow muscle cells are used primarily for the slow undulatory swimming necessary for locomotion and feeding. They are also involved in stereotypic movements called “coiling.” Coiling involves slow spontaneous contractions by zebrafish embryos around 24 hpf. These movements are dependent solely on slow fibers; when fast fibers are functionally absent, coiling can still be observed [41]. In contrast, fast muscle cells are used for the powerful C-bend contraction and burst swimming needed to escape from adverse and potentially dangerous stimuli.

To meet these functional requirements, slow and fast muscle cells utilize AChRs with the aforementioned different subunits and kinetics. Slow muscle cells have fivefold slower synaptic decay times in order to sustain longer slower contractions. Since slow fibers in zebrafish lack action potentials, it is possible that slower synaptic current kinetics are necessary to ensure effective muscle contractions. Indeed, it was previously shown that slower decaying synaptic currents in the developing soleus muscle of rats gives rise to contractions whereas faster currents do not [42].

Fast muscle cells, on the other hand, utilize both action potentials and very fast synaptic currents to generate quick powerful contractions [34]. Slowing the kinetics of synaptic input can have profound effects on the locomotion and health of the fish. For instance in the zebrafish mutant *twister*, which has a mutation in the 2nd transmembrane region of the AChR α subunit, channel openings are prolonged and synaptic currents are delayed. As a result, muscle contractions are so protracted and powerful to the extent that they actually prevent locomotion. Not surprisingly, the *twister* mutation is lethal in homozygous fish embryos [32].

## “Slow” muscle fibers in mammalian versus non-mammalian vertebrates

The term “slow muscle” has been used to refer to muscle fibers across vertebrates. However, the properties of these slow muscles vary significantly between species. In Table 1, some of the characteristics of slow fibers across species are summarized.

**Table 1:** Comparison of slow muscle fiber characteristics

	“Tonic” fiber in frog	Slow fiber in (zebra) fish	Mammalian AP-less fiber	Mammalian type I fiber
Innervation	En grappe/myoseptal	Myoseptal	Multiple innervation	En-plaque
Action potential	(−)	(−)	(−)	(+)
Synaptic current kinetics	Slow	Slow	NA	Slow?
AChR subunits	NA	αβδ	NA	NA

We propose that these “slow” fibers could be divided into two distinct groups on the basis of their spiking ability: slow fibers without action potentials (AP-less fibers; 1st–3rd columns in Table 1) and mammalian type I fibers which exhibit action potentials (type I fibers; 4th column in Table 1).

AP-less fibers include tonic fibers in frog [3, 23, 43–47], slow fibers in fish [22, 24, 34], and fibers in mammalian extraocular muscles [48, 49] [27, 50].

Common properties found in the AP-less fibers include the lack of action potentials [2, 50, 51], multiple nerve endings [47, 52] [24, 53], and possibly slow synaptic current kinetics at the neuromuscular junction [34, 47]. These fibers correspond largely to what Morgan and Proske called “true slow fibers”, which conform more to the classical definition of “tonic” fibers and are “much more widely distributed in muscles of submammalian vertebrates” [14]. Properties found in these fibers may be functionally linked: the lack of action potentials may necessitate synaptic currents with slower kinetics that initiate from multiple locations along a single fiber in order to evoke effective depolarization and contraction. Whether different compositions of AChRs observed in zebrafish underlie synaptic properties of slower current kinetics, for example in mammalian extraocular fibers, awaits further studies.

Mammalian type I fibers have action potentials and en-plaque type synapses [14]. Bewick et al. showed that muscle fibers in rat soleus muscle have somewhat slower decays of miniature endplate potentials (mEPPs), which may reflect AChR kinetics, than those in the extensor digitorum longus (EDL) at certain developmental stages [54]. The soleus muscle contains a proportion of type I fiber as high as 40 %, while the EDL muscle is composed mostly of type II fibers [55]. This suggests that mammalian type I fibers may also have slower decaying synaptic currents compared to type II fibers, though the difference is not as large as seen in AP-less fibers. Further studies of neuromuscular junctions in type I muscles will provide more information.

## Conclusion

Electrophysiological and molecular studies of zebrafish neuromuscular transmission have revealed surprising synaptic properties unique to slow muscle fibers. Results from these studies suggest that the kinetics and AChR subunit composition of NMJ synapses may help better define AP-less fibers. Ultimately, these findings need to be tested in other vertebrate species in order to establish a global categorization of slow muscle fibers encompassing AP-less fibers and mammalian type I fibers. Such a generalizable definition is essential to the conceptual framework of skeletal muscle physiology that serves as the basis for present and future translational applications.
